# CognitionMaster: an object-based image analysis framework

**DOI:** 10.1186/1746-1596-8-34

**Published:** 2013-02-27

**Authors:** Stephan Wienert, Daniel Heim, Manato Kotani, Björn Lindequist, Albrecht Stenzinger, Masaru Ishii, Peter Hufnagl, Michael Beil, Manfred Dietel, Carsten Denkert, Frederick Klauschen

**Affiliations:** 1 Institute of Pathology, Charité University Hospital Berlin, Charitéplatz 1, Berlin, 10117, Germany; 2 VMscope GmbH, Charitéplatz 1, Berlin, 10117, Germany; 3 Laboratory of Cellular Dynamics, WPI-IFReC, Osaka University, 3-1 Yamada-oka, Suita, Osaka, 5650871, Japan; 4 CREST, Japan Science and Technology Agency (JST), 5 Sanbancho, Chiyoda-ku, Tokyo, 1020075, Japan; 5 University of Applied Sciences Berlin, Wilhelminenhofstraße 75A, Berlin, 12459, Germany; 6 Institute of Pathology, University Hospital Heidelberg, Im Neuenheimer Feld 220/221, Heidelberg, 69120, Germany; 7 Department of Medicine I, University of Ulm, Albert-Einstein-Allee 23, Ulm, 89081, Germany

**Keywords:** Software, Open source, Image analysis, Object-based image analysis

## Abstract

**Background:**

Automated image analysis methods are becoming more and more important to extract and quantify image features in microscopy-based biomedical studies and several commercial or open-source tools are available. However, most of the approaches rely on pixel-wise operations, a concept that has limitations when high-level object features and relationships between objects are studied and if user-interactivity on the object-level is desired.

**Results:**

In this paper we present an open-source software that facilitates the analysis of content features and object relationships by using objects as basic processing unit instead of individual pixels. Our approach enables also users without programming knowledge to compose “analysis pipelines“ that exploit the object-level approach. We demonstrate the design and use of example pipelines for the immunohistochemistry-based cell proliferation quantification in breast cancer and two-photon fluorescence microscopy data about bone-osteoclast interaction, which underline the advantages of the object-based concept.

**Conclusions:**

We introduce an open source software system that offers object-based image analysis. The object-based concept allows for a straight-forward development of object-related interactive or fully automated image analysis solutions. The presented software may therefore serve as a basis for various applications in the field of digital image analysis.

## Background

Image analysis has been an active research field since the development of scientific computing decades ago and since then many biomedical (and other) studies have been reported that rely on image analysis software to support their investigations, e.g. [1-6] just to name a few. Several open source imaging projects exist in that context with *ImageJ*[7] as one of the most popular. Some other projects, e.g. *CellProfiler*[8], *Fiji*[9] and *icy*[10] are based on *ImageJ* and provide powerful and flexible user interfaces including region-of-interest (ROI) selection, data analysis and plug-in interfaces. Other software frameworks like *OpenCV*[11] and *ITK*[12] are designed as libraries that provide algorithms for the integration into high-level applications. A common feature of the aforementioned solutions is that the basic processing unit is the single image pixel, which is the smallest processable unit of a digital image containing intensity and color information (see [13] for the measurement of image information). By grouping pixels into distinct segments (referred to as objects) using the pixel intensity/color information or image texture biological meaningful objects and structures may be represented and used for an object-based analysis [14-16]. This can be advantageous when object-related properties (features) and relationships between objects (neighbourhood) have to be taken into account. As an example: After the initial segmentation of certain image objects (e. g. cells) a typical task in image analysis is to classify all objects according to certain features (e. g. dark and bright objects). With our approach the user may easily experiment interactively with the classification step by computing various features (e.g. size, shape localization and color) and then study correlations between different properties to define the optimal classification routine. This is all done on the object level and connected with visualization of feature statistics. Another important question is the topological relationship between objects of the different classes. Evaluating topological problems could in principle be solved with the aforementioned pixel-based programs, too, but its implementation becomes less complicated if the basic processing unit is the image-object. Unfortunately, open source software tools that utilize object-based image analysis and cover a broad spectrum of functionality comparable to the flexible pixel-based tools mentioned above are not yet available. Here, we present an object-based open-source software platform that may be used for a broad spectrum of tasks in the field of (biomedical) image analysis: Ranging from algorithm development with an integrated C# compiler to (pipeline-style) one-click analysis provided through a powerful plug-in interface. An object layer structure was designed to handle image objects and their properties and therefore allow high-level formulations of image analysis algorithms. The tool provides flexible and interactive functionality with a variety of image analysis algorithms that may be combined in process chains, an object model editor and visualization/statistics functions. We demonstrate the utility of our approach with the design of an exemplary processing chain for quantifying Ki67 proliferation marker [17,18] in bright-field histological breast cancer. A second example shows the advantages of the object-based software design for the analysis of relationships between objects for the quantification of osteoclast–bone interactions in two-photon fluorescence microscopy data [19,20].

### Implementation

**Figure 1:**
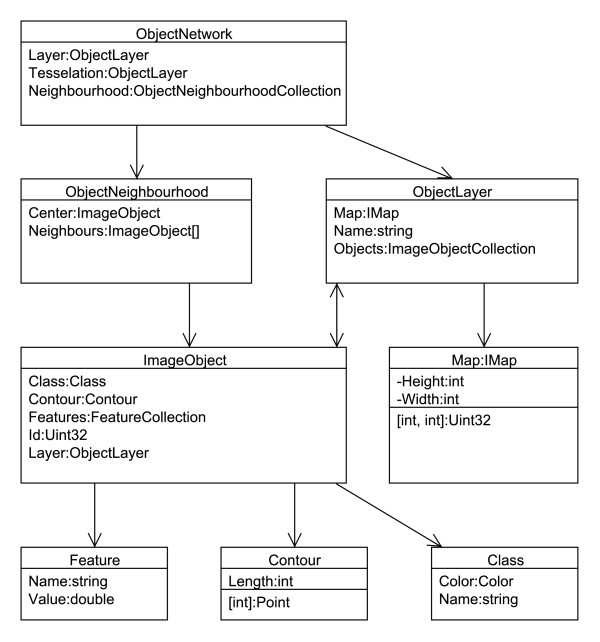
**Excerpt of the class diagram showing the main data structures used for the object-based imaging: the *ImageObject *class summarizes all object-related properties (e.g. classification, contour, features).** All objects (of a certain processing step) built an *ObjectLayer*.

**Figure 2:**
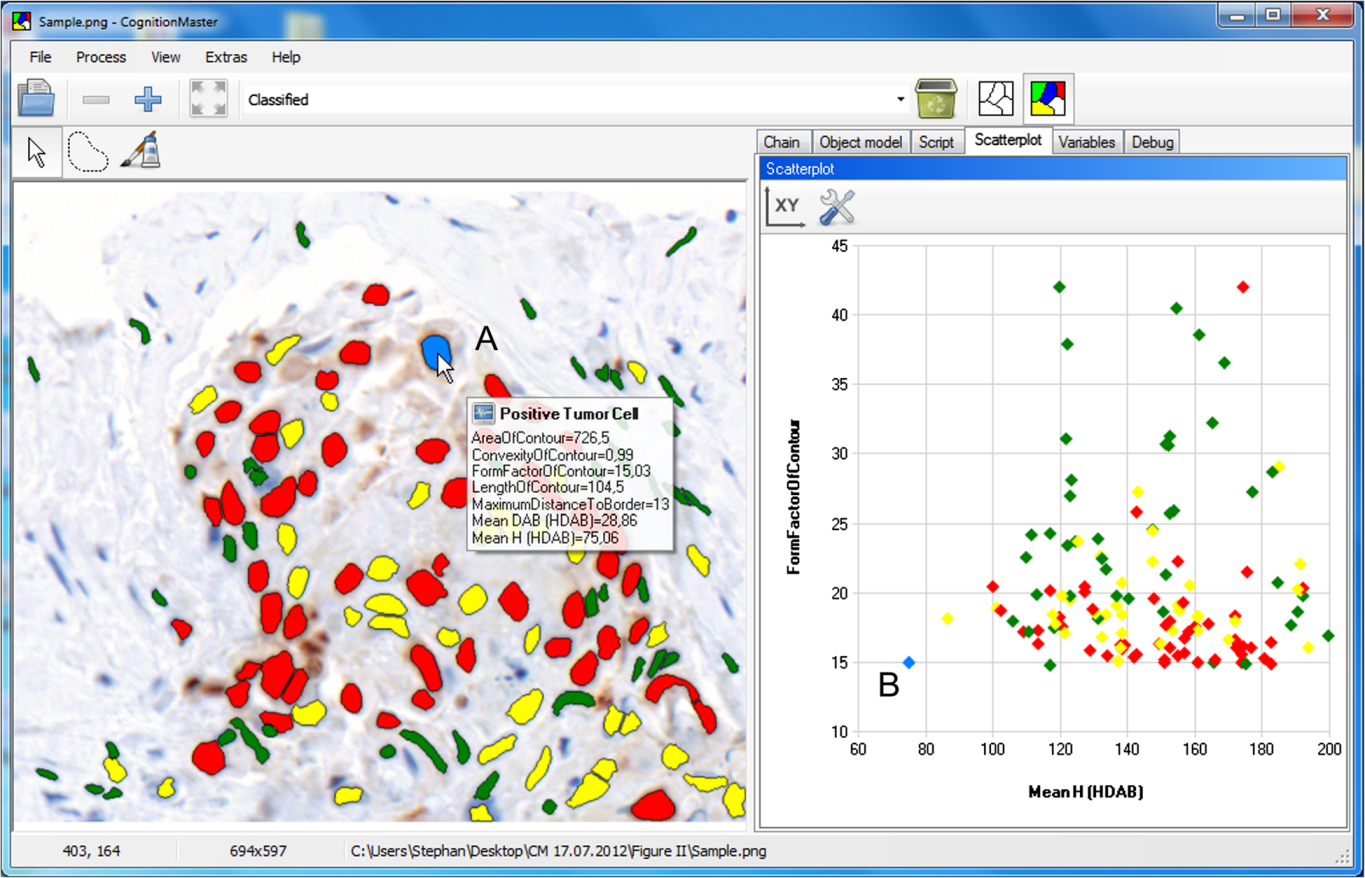
**Image of Ki67 **[18]** stained breast cancer tissue displayed in *CognitionMaster *with Ki67+ tumor cells (red), Ki67- tumor cells (yellow) and normal cells (green).** Segmentation was performed according to [28]. **A**. Showing the objects features as a mouse-over effect. **B**. Scatter plot diagram displaying two features for all image objects (cells) marked in the image on the left: the mean haematoxylin intensity in the H-DAB stain (x-axis) and a form factor given by contour-length^2^/contour-area (y-axis). The displayed features may be selected by the user once the features are computed (*Process* menu). **A** and **B**. Objects may be selected in the image or in the scatter plot diagram. Selected objects are highlighted in both presentations (blue).

*CognitionMaster* was implemented in *C#* for *Microsoft.NET*. *SharpDevelop*[21] was used for the integrated editing of *C#* code. Icons from the *Tango!*[22] project were used for the graphical user interface. A documentation of the application programming interface (API) was generated using *doxygen*[23]. Figure 1 shows the main data structures, which were designed to organize objects/segments (instead of image pixels) and their properties and therefore allow for an object-based (with respect to handle image contents) modeling of software applications and algorithms. The class *ImageObject* represents a coherent group of neighbouring pixels (segment). Several properties can be used to describe an *ImageObject* as the contour for its spatial position or a collection of key value pairs (*Features*) for properties like mean intensity, object pixels etc. The *ObjectLayer* class was designed to summarize all information of one processing step: it contains a list of all *ImageObjects* and a two-dimensional map of the same size as the corresponding image which contains the identifier of the *ImageObject* a pixel belongs to. This map may be used for pixel-based access to objects or pixel-based operations (e.g. distance transformation). In an analysis pipeline sequential processing modules obtain and pass on their input as *ObjectLayer* instances containing all objects and the respective features. Discrete Voronoi tessellation [24] on the object layers map is used to compute the topological relationships. These are represented in an *ObjectNetwork* class which contains the *ObjectNeighbourhood* for each object. The neighbourhood information can be used, e.g. to compute the length of the border to an object of a certain class or the number of neighbouring objects (or touching objects, respectively) of a certain class. The object-based design makes it easier to communicate between algorithms and handle user interactions: instead of passing a label image (were often the meaning of the labels is implicit) and several lists/dictionaries containing the related properties only an object layer or even a single object has to be passed. E.g. when the mouse is moved over a certain object the *MouseEnterObject* and *MouseLeaveObject* events are fired. The corresponding object is passed with the events data. Via this object reference all consumers of the event get access to all object properties and the corresponding object layer. The *MouseEnterObject* event, for example, is used to display the classification and features of this object (Figure 2A). Everything that happens is that in the event handler the name of the assigned class and the objects features are read from the corresponding *ImageObject* instance and printed to the popup. The object-based approach also allows for the (multi-) selection of objects in the image and scatter plot diagram. Selected objects are highlighted in both representations (Figure 2A and B). All algorithms can be used within the graphical user interface or in any other .NET application by referencing the *CognitionMaster* assemblies.

## Results

**Figure 3:**
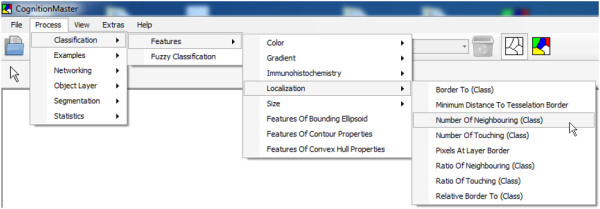
**The *Process *menu of the main application window.** This menu is generated on the applications startup: Therefore all available scripts and processing chains are loaded. Users may add own scripts to the respective directory resulting in additional menu entries.

**Figure 4:**
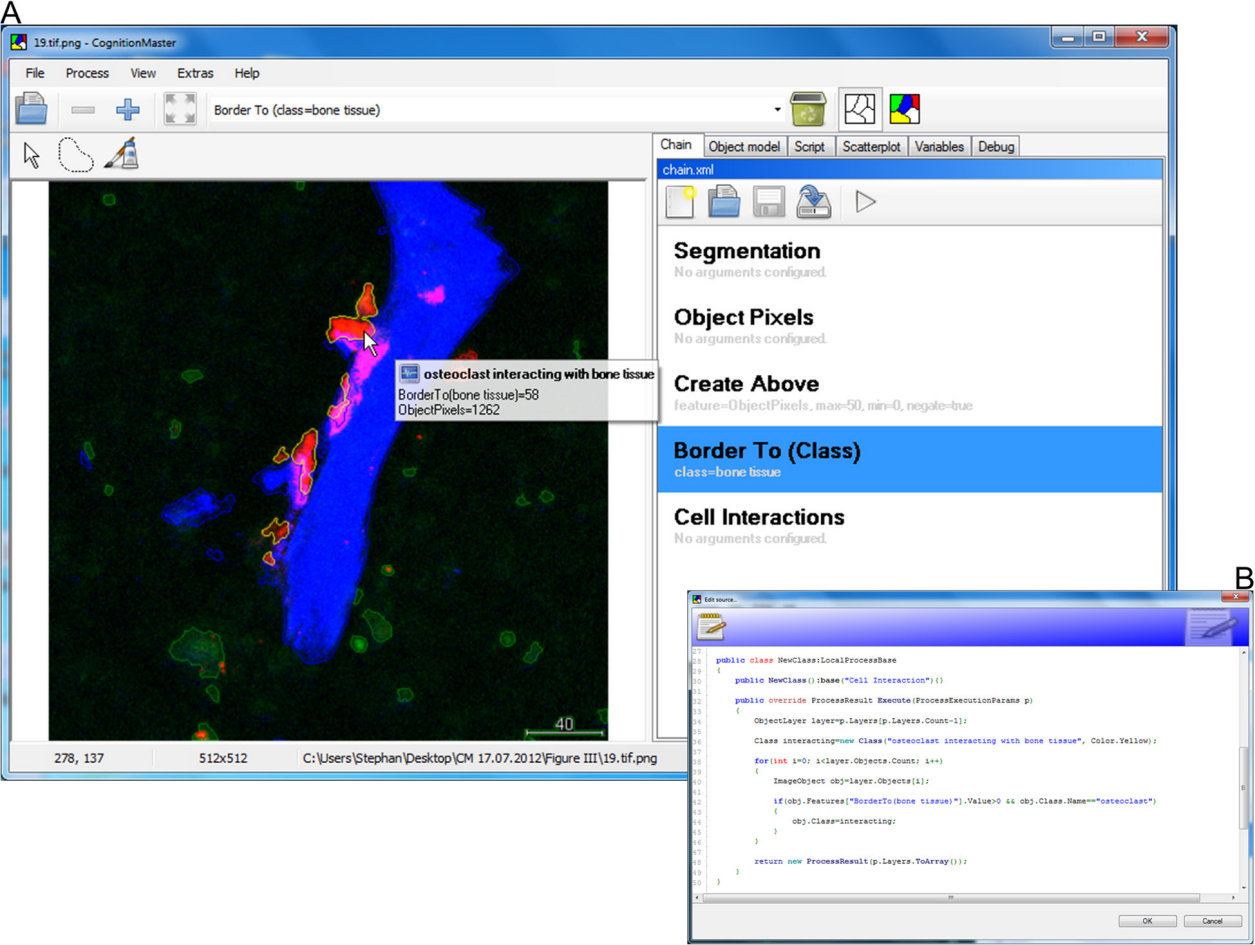
**User defined cell interaction analysis of a fluorescent bone image with in-program scripting. A**. Processing chain with standard elements (e.g. the computation of the length of the objects border to objects classified as bone tissue) and user-defined in-program scripts (e.g. the segmentation and (primary) classification of the three object types bone tissue (blue), osteoclast (green) and monocyte (red)). Names of processing steps are displayed in large black letters, configured arguments in small gray letters. Segmentation result with red, green and blue contours. Interacting cells with yellow contours. **B**. Source code editor showing the source code of the “Cell Interactions” processing step that computes the length of the border of osteoclasts to bone tissue and assigns the “osteoclast interacting” class if this length is above zero.

*CognitionMaster* is a software tool designed to serve as a basis for various interactive and/or fully automated image analysis tasks. The plug-in concept allows for the expansion of the software with new menu entries, tab pages and handling of special image formats, e.g. plug-ins for the support of DICOM ^a^and whole slide images ^b^(WSI) are available from the download page. Plug-ins even may extend the image rendering to present additional image (meta) information e.g. image sharpness maps [25] or visited regions [26] in virtual slides, an emerging field of medical imaging [27]. Additionally, plug-ins may change the default behavior of the software (e.g. reactions on mouse and key events) by overriding the corresponding event handler: the ROI manager plug-in enables users to assign labels to points or regions of interest by mouse-clicks. Besides the application *CognitionMaster* offers a *C#* component library with more than 250 classes (image analysis algorithms, data structures and visual components, etc.). Image analysis algorithms may be selected from the *Process* menu (Figure 3), whereas results of prior executed algorithms may be used as input for consecutive processing steps. Processing steps may also be combined into processing chains (“analysis pipelines”). Objects are rendered as contours or filled, colored according to an assigned class (Figure 2 and 4) or with a default color if no class is assigned. A debug console may be used to output status messages when writing new algorithms (see in-program scripting section for details). For global data interchange (between plug-ins and/or in-program scripts) the *VarInterop* plug-in may be used: this plug-in manages and displays global variables. For data visualization a two-dimensional interactive scatter plot diagram is available. How objects can be classified best can be tested using the built-in object model editor: This plug-in enables the user to describe the ideal “model” object with features. This object model may be applied to a certain image and the user may check if his “object model” meets the real image.

### Ki67 proliferation assessment in breast cancer immunohistology samples

In the following section we demonstrate how different methods or hypothesis can be tested using a processing chain for the quantification of proliferation marker assessment in breast cancer using Ki67 immunhistochemic staining [17,18]. Scoring proliferation through Ki67-immunohistochemistry is an important component in predicting response to chemotherapy in breast cancer patients. Therefore, positive and negative stained tumor cells have to be detected and counted by the pathologist in routine diagnostics. If one would like to set up a fully automated scoring system 4 major functions have to be implemented:

a. Detection and segmentation of cell nuclei

b. Classification into tumor and non-tumor cells

c. Classification into Ki67-positive (Ki67+) and Ki67-negative (Ki67-) cells

d. Counting of all Ki67+ and Ki67- tumor cells

A method for the detection and segmentation of cell nuclei (a) was introduced recently in [28]. The modules described in this study (contour-based segmentation, non-compact pixel removal and concave object separation) are standard-components of the *CognitionMaster* library. For the second step of the scoring system (b) it may be interesting to test several hypothesizes on how tumor cells can be distinguished best from non-tumor cells: often non-tumor cells are smaller than tumor-cells or have a different aspect ratio (connective tissue cells are often spindle-shaped). For the validation of these hypothesizes it is important to be able to easily get an impression of the feature values of a certain object or the distribution of a certain feature respectively. Therefore, users may select one or more features from the *Process* menu once the segmentation is executed. The selected features are computed for each object (cell nuclei). Then, features are displayed in a popup when the mouse cursor is moved over a certain object (Figure 2A). Additionally, the scatter plot diagram (Figure 2B) can be used to decide which feature and threshold is optimal. For the classification into Ki67+ and Ki67- cells features based on color deconvolution [29] may be used from the *Immunohistochemistry* group. Finally, the number of cells for each class are counted using *Num(Class)* algorithm available from the *Process* menu. The complete example including the sample image, the processing chain and a guidance file (readme.txt) can be found in Additional file 1 online.

### Cell interaction analysis

In the example described here, we demonstrate the advantages of our object-based approach for the analysis of relationships between objects. Therefore, we use image analysis to test the hypothesis that osteoclast attachment to bone surface as a proxy for bone decomposition is influenced by sphingosine-1-phosphate [19]. The main aspect of this approach is the analysis of interactions between bone tissue and osteoclasts. *CognitionMaster* contains a set of routines/algorithms that allow for the computation and analysis of object neighbourhoods e.g. the *ObjectNetwork* class (Figure 1) and the *NumberOfNeighbouring* and *NumberOfTouching* features. The first step of the analysis pipeline is the detection of the different types of tissue/cells (bone tissue, osteoclasts, monocytes). Here we re-implemented the detection algorithm including the threshold selection method and channel merging approach presented in the original publication [20] using in-program scripting (Figure 4, see in-program scripting section and the first processing step in the processing chain in Additional file 2 online). After the segmentation the next step of the analysis is the computation of the interfacing-length between bone tissue and osteoclasts. This can easily be solved by using the *BorderTo* feature, which is a standard component of the software and computes the number of contour pixels with a neighbouring pixel of another object class. Finally, the user may export the results containing the object id, class name and features into a .csv file which he may then open with other statistics tools (e.g. Microsoft EXCEL) for further analysis. The complete example containing the sample image, the processing chain and a guidance file (readme.txt) can be found in Additional file 2 online.

### In-program scripting

While many image analysis algorithms are already available, the development of new methods is an important field in digital image analysis. Furthermore many image analysis tasks require special pixel- or object-based operations that can hardly be part of common purpose image analysis software e.g. the specialized automatic threshold segmentation and channel merging technique used in the previous section. It can be very helpful to be able to write and execute user-specific source code without needing an extra software project or switching between different applications. A high-execution speed is important while testing the new idea and in the consecutive deployment to other users which should be, however, as simple as possible: it should be possible to use the new code directly among other (standard) components without the need for a re-implementation due to performance or deployment reasons. *CognitionMaster* comes with an integrated C# editor/compiler: this component enables the user to write, compile and execute standard C# code (so called scripts) within the main application (Figure 4B). For most algorithms contained in the *CognitionMaster* library (e.g. watershed-segmentation and extraction of various features) a script comes with the installation and can be accessed via the *Process* menu (Figure 3), which is generated dynamically when the software is launched: Therefore all available scripts and processing chains are read from a certain file directory. This offers the opportunity to add own scripts to this menu. A major advantage of the in-program scripting technique is the high execution speed: the script is compiled using the standard *.NET* compiler and then executed with the same performance as a normal *.NET* executable (not an interpreted script). In contrast, the macro language of *ImageJ* is interpreted and therefore offers lower performance. Additionally, scripts can be combined in processing chains, whereby the source code of a script is copied into the processing chain and compiled when executing. Embedding the source code (instead of linking to it) offers the possibility to change the behavior (source code) of standard components in a certain processing chain. Furthermore, arguments can be denoted for each processing step, which avoids the necessity of using input dialogs at each module.

### Comparison with ImageJ

With *ImageJ* the built-in *ROI Manager* and *Particle Analyzer* can be used to analyze images on the object-level (see Additional file 3 online for details). Therefore a binary image is required that marks every pixel either as foreground or background. Unfortunately, differentiating two touching objects is not possible with this technique, which is a major drawback compared to our approach. Once the particles are found objects are shown as an overlay. Unfortunately, the user has to select preliminary how objects are rendered (e.g. object contours, ellipses or filled). It is not possible to easily switch “online” between different rendering modes. In contrast, *CognitionMaster* allows “online” switching of the rendering mode (contours, filled, none) and moreover, supports visualization of classification results by rendering each object with the color of the assigned class. *ImageJ* only allows for the selection of certain objects by using the object list provided by the *ROI Manager* plug-in. Free-hand selection or selecting individual objects by mouse-clicks are not supported. Mouse-over feature statistics for a certain object are also not supported by *ImageJ* as well as the interactive scatter plot diagram, which are both available in *CognitionMaster* (see Figure 2). Finally, *CognitionMaster* allows for overriding or extending user-interactions by using the corresponding events (e.g. *MouseEnterObject*, *MouseLeaveObject* or *SelectedObjectsChanged*), which is not possible with *ImageJ* because of the lack of a dedicated object model.

## Discussion

Conventional image analysis applications were designed with the single image pixel as basic processing unit. This concept is advantageous when image-to-image transformations (filters) are the main purpose of the software application, but has limitations when image objects and their properties and relationships between objects are considered. In this case the object-based approach allows for the use of high-level features, such as the interactivity with image objects like mouse-over effects (Figure 2A) and data-analysis on the object level through a GUI-based pipeline assembly such as, for instance, the evaluation of spatial relationships between cells. Object-based approaches, however, require *a priori* object segmentation/detection, for which the *CognitionMaster* offers a variety of established approaches and the option to implement alternative methods using in-program scripting. Moreover, using object-based data structures to process object-based image contents facilitates the design of a software architecture compliant to state-of-the-art object-oriented-programming (OOP). This favors clarity of design and especially the reusability of available analysis modules not only in processing pipelines or in-program scripts within the *CognitionMaster,* but also as components in other .NET projects. In contrast to that, several label maps (or one label map and several lists) are required to store “pixel” to “segment”, “segment” to “features” and “segment” to “class” mappings in pixel-based approaches, which can hardly be reused (e.g. by inheritance).

## Conclusions

We introduce the open source software system *CognitionMaster* that features object-based image analysis and implements a large variety of image analysis algorithms that may be extended using a plug-in interface or in-program scripting. Therefore, *CognitionMaster* may serve as a basis for a broad range of applications in the field of digital image analysis.

### Availability and requirements

*CognitionMaster* is released under the terms of the GNU General Public License (GPL). The installation packages, source code files and documentation for the main application and available plug-ins can be found on http://sourceforge.net/projects/cognitionmaster/. *CognitionMaster* can be installed on all *Microsoft Windows* PCs (*Windows XP* or newer, 32-bit or 64-bit). *Microsoft .NET* 2.0 is required. A documentation of the application programming interface (API) is provided with the installation packages.

## Endnotes

^a^Using ClearCanvas, free

^b^Using Virtual Slide Access – SDK 4.0 from VMscope GmbH (Germany), commercial

## Competing interests

The authors declare no conflict of interests.

## Authors’ contributions

SW, FK and DH wrote the paper. SW implemented the software. BL wrote the software manual. FK, AS, CD, PH, MD and MB supervised the software design and architecture and algorithm implementation. DH, AS and FK performed software tests. FK and CD selected and provided histological samples. MI and MK provided the two-photon microscopy data and advised on the image analysis. All authors reviewed the manuscript. All authors read and approved the final manuscript.

## Supplementary Material

Additional file 1 Example 1. Click here for file

Additional file 2 Example 2. Click here for file

Additional file 3 Comparison with ImageJ. Click here for file
