# Supplementary figures and images for: CognitionMaster: an object-based image analysis framework
Source: Diagn Pathol. 2013 Feb 27;8:34. doi: 10.1186/1746-1596-8-34 (PMC3626931; doi:10.1186/1746-1596-8-34)

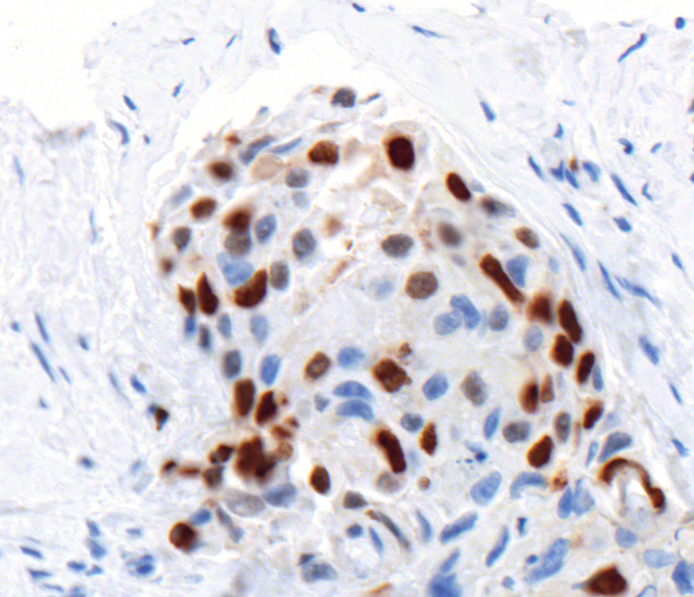

Supplement: Additional file 1 — Example 1. [file 1746-1596-8-34-S1.zip › Additional File 1/Sample.png]

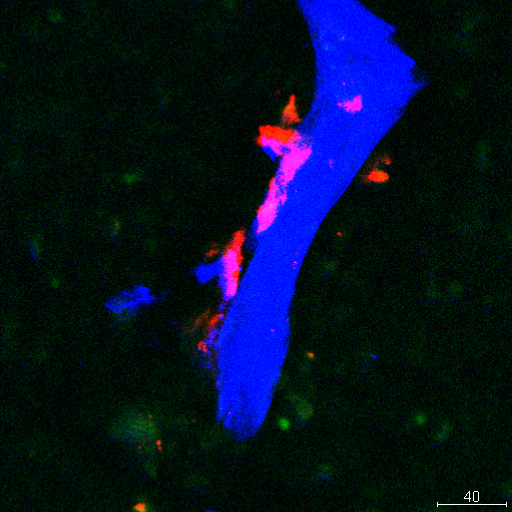

Supplement: Additional file 2 — Example 2. [file 1746-1596-8-34-S2.zip › Additional File 2/19.tif.png]
