# Supplementary material for: CognitionMaster: an object-based image analysis framework
Source: Diagn Pathol. 2013 Feb 27;8:34. doi: 10.1186/1746-1596-8-34 (PMC3626931; doi:10.1186/1746-1596-8-34)
Supplement: Additional file 3 — Comparison with ImageJ. [file 1746-1596-8-34-S3.docx]

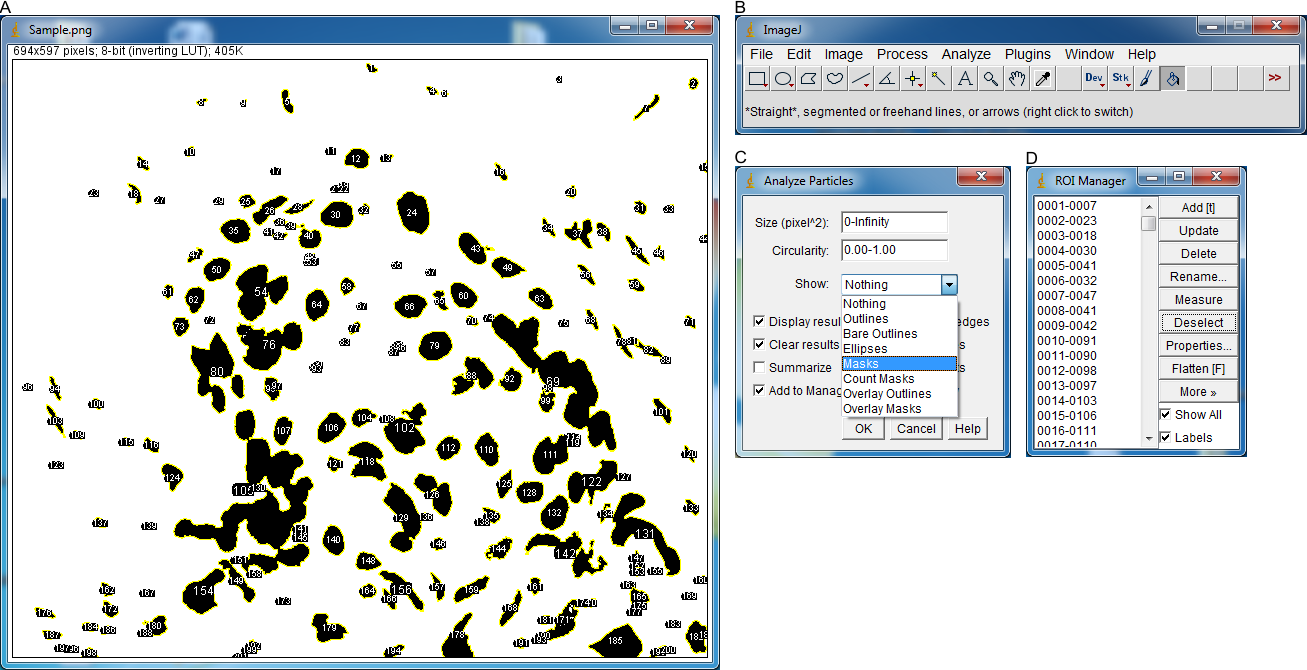


Figure 1: Analysis on the object level with *ImageJ*. A. The binary image with foreground pixels (black) and background pixels (white). B. The *ImageJ* main window. C. The options window of the particle analyzer plug-in. The object presentation (drop down list) has to be selected preliminary. D. The main window of the *ROI Manager* plug-in. Objects may be selected only by using this object list.
